# Blocking insulin-like growth factor 1 receptor signaling pathway inhibits neuromuscular junction regeneration after botulinum toxin-A treatment

**DOI:** 10.1038/s41419-023-06128-w

**Published:** 2023-09-16

**Authors:** Hiroki Ishihara, Yoshinori Otani, Kazuki Tanaka, Hisao Miyajima, Huy Xuan Ngo, Masashi Fujitani

**Affiliations:** 1 Department of Anatomy and Neuroscience, Faculty of Medicine, Shimane University, 89-1 Enya-cho, Izumo-shi, Shimane 693-8501 Japan; 2 Department of Rehabilitation, Faculty of Medicine, Shimane University, 89-1 Enya-cho, Izumo-shi, Shimane 693-8501 Japan; 3 Department of Oral and Maxillofacial Surgery, Faculty of Medicine, Shimane University, 89-1 Enya-cho, Izumo-shi, Shimane 693-8501 Japan

**Keywords:** Somatic system, Molecular neuroscience

## Abstract

Botulinum toxin-A (BTX) administration into muscle is an established treatment for conditions with excessive muscle contraction. However, botulinum therapy has short-term effectiveness, and high-dose injection of BTX could induce neutralizing antibodies against BTX. Therefore, prolonging its effects could be beneficial in a clinical situation. Insulin-like growth factor-1 receptor (IGF1R) and its ligands, insulin-like growth factor (IGF) -I and II, regulate the physiological and pathological processes of the nervous system. It has been suggested that IGF1R is involved in the process after BTX administration, but the specific regeneration mechanism remains unclear. Therefore, this study aimed to determine how inhibition of IGF1R signaling pathway affects BTX-induced muscle paralysis. The results showed that anti-IGF1R antibody administration inhibited the recovery from BTX-induced neurogenic paralysis, and the synaptic components at the neuromuscular junction (NMJ), mainly post-synaptic components, were significantly affected by the antibody. In addition, the wet weight or frequency distribution of the cross-sectional area of the muscle fibers was regulated by IGF1R, and sequential antibody administration following BTX treatment increased the number of Pax7^+^-satellite cells in the gastrocnemius (GC) muscle, independent of NMJ recovery. Moreover, BTX treatment upregulated mammalian target of rapamycin (mTOR)/S6 kinase signaling pathway, *HDAC4*, *Myog*, *Fbxo32/MAFbx/Atrogin-1* pathway, and transcription of synaptic components, but not autophagy. Finally, IGF1R inhibition affected only mTOR/S6 kinase translational signaling in the GC muscle. In conclusion, the IGF1R signaling pathway is critical for NMJ regeneration via specific translational signals. IGF1R inhibition could be highly beneficial in clinical practice by decreasing the number of injections and total dose of BTX due to the prolonged duration of the effect.

## Introduction

Botulinum toxin-A (BTX) is an established treatment modality for conditions with excessive muscle contraction, such as dystonia, spasticity, infantile cerebral palsy, hemifacial spasm, tics, tremor, and motility disorders of the bladder and gastrointestinal tract [1, 2]. However, botulinum therapy has short-term effectiveness and thus requires repeated administration. High-dose injection of BTX could induce neutralizing antibodies against BTX, significantly reducing its effectiveness [3, 4]. Therefore, decreasing the number of injections and the total dose of BTX by prolonging its effect will significantly benefit clinical practice.

The neuromuscular junction (NMJ) is a specialized synapse between motor nerve endings and their muscle fibers, where electrical impulses generated by neurons are converted into electrical activity in muscle fibers [5]. BTX is incorporated into the presynaptic terminal by endocytosis and cleaves the synaptic target protein synaptosomal-associated protein 25, which inhibits acetylcholine release and induces paralysis [6, 7]. The formation of sprouting fibers from proximal axons in the main NMJ results in a small NMJ in the periphery, eventually regenerating the main NMJ and resulting in spontaneous recovery from muscle paralysis [8]. However, the specific molecular mechanism remains unclear in BTX-induced muscle paralysis.

Insulin-like growth factor-1 receptor (IGF1R) and its ligands, insulin-like growth factor (IGF) -I and II play critical roles in physiological and pathological processes [9–11]. As a nerve growth factor in the skeletal and nervous systems, the IGF1R signaling pathway regulates brain development [12], synaptogenesis [13], neuroprotection [14], nerve regeneration [15], and skeletal muscle growth and differentiation [16]. The involvement of IGF-I, IGF-II, and the IGF1R signaling pathway in skeletal muscle cell regulation has been well described [17]. Muscles express both IGF-I and IGF-II upon injury [18], and IGF-I activates satellite cells (SCs) and promotes skeletal muscle regeneration [17]. IGF-I has also been shown to increase the synthesis of skeletal muscle protein via the phosphoinositide 3 kinase/Akt/mammalian target of rapamycin (PI3K/Akt/mTOR) pathway and to counteract muscular proteolytic pathways (e.g., ubiquitin-proteasome system) and autophagy [17]. Moreover, a single base mutation in the third intron of IGF-II increased muscle mass by upregulating IGF-II transcript in domestic pigs [19] and knock-in mice [20]. In addition, IGF-I and IGF-II activate the PI3K/Akt/mTOR pathway to increase myogenesis and IGF-II orchestrates the development of fast myofibers [21]. Furthermore, IGF-I, IGF-II, and IGF1R signaling pathway is involved in several aspects of neurological and skeletal disorders, such as nerve degeneration [22], muscle aging [23], sarcopenia [24], thyroid ophthalmopathy [25], and autoimmune diseases [26].

IGF-I play critical roles at the NMJ [27]. Particularly, IGF-I inhibits aging-related dysfunction and morphological abnormalities of the NMJ [24, 28]. Further, local expression of IGF-I in the skeletal muscle has been found to inhibit motor neuron degeneration and NMJ dysfunction in an amyotrophic lateral sclerosis mouse model [29]. Moreover, IGF-I systemic expression in a spinal muscular atrophy mouse model has been shown to improve NMJ abnormalities [30]. Aged regenerating nerves exposed to peripheral nerve injury are sensitive to IGF-I treatment [15, 31].

Taken together with these results, we hypothesized that the IGF1R signaling pathway plays a critical role in muscle denervation by BTX. In this study, we aimed to investigate the effect of suppression of the IGF1R signaling pathway in BTX-induced muscle paralysis and thus analyzed the morphological changes in the NMJ, sprouting fibers, and the differentiation of SCs.

## Materials and methods

### Reagents and animals

General reagents not otherwise specified were guaranteed reagent grades from Wako Pure Chemical Co. (Osaka, Japan), Nacalai Tesque (Kyoto, Japan), and Sigma-Aldrich Japan/Merck (Tokyo, Japan).

Male B6.Cg-Tg(Thy1-YFP)16jrs/j mice (Thy1-YFP mice) (The Jackson Laboratory, Bar Harbor, ME, USA) [32] and C57BL/6 J mice (SLC Co., Shizuoka, Japan) aged 6–8 weeks were used in this study. Thy1-YFP mice were used for analysis of the NMJ and C57BL/6J mice were used for muscular and SCs analysis. The animals were reared at the Animal Experimentation Department, Research Support Center for Integrated Science Research, Shimane University Research Organization. Breeding conditions were set at a room temperature of 23 ± 2 °C, humidity of 55 ± 10%, ventilation frequency of 10–13 times/h on average, and light-dark switching conditions (light period, 7:00–19:00; dark period, 19:00–7:00) every 12 h for proofing.

This study was approved by the Animal Experimentation Committee as per the Animal Experimentation Guidelines of the Shimane University Research Support Center for Integrative Science (IZ31-57, IZ31-58, IZ2-123, and IZ4-11) and ARRIVE guidelines.

### Intramuscular administration of anti-IGF1R antibody in the gastrocnemius (GC) muscle

A BTX stock solution was prepared by diluting Botox© 100 U (Allergan, Dublin, Ireland) to 0.1 U/µL of 1 mL saline. Stock solutions were diluted to 1.25 and 2.5 U concentrations. Dilutions were performed using saline with 0.005% Fast Green FCF (Sigma-Aldrich Japan/Merck) for dose site confirmation. The mice were anesthetized by intraperitoneal administration of an anesthetic mixture of three reagents (0.3 mg/kg medetomidine [Fujita Pharmaceutical Co., Tokyo, Japan], 4.0 mg/kg midazolam [Maruishi Pharmaceutical Co., Osaka, Japan], and 5 mg/kg butorphanol [Meiji Seika Pharma Co., Tokyo, Japan]).

The dorsal surfaces of both the hindlimbs were shaved, and the skin at the injection site was incised to ensure that the fascia was visible. BTX (50 µL) was administered intramuscularly to the right GC muscle of the mice, and 50 µL saline was administered to the left GC muscle using a manipulator (UM-1C/IM-3) (Narishige Scientific Instruments Laboratory, Tokyo, Japan) and a Hamilton syringe (710RN) (Hamilton, Reno, NV, USA), respectively. For the single-dose experiments, 2.5 U BTX and 20 µg IGF1R monoclonal antibody or 20 µg mouse IgG1 as an isotype control were prepared to a total volume of 50 µL and administered into the right GC muscle.

For the repeated antibody administration experiment, 1.25 U BTX and 20 µg IGF1R monoclonal antibody or 20 µg mouse IgG1 were prepared to a total volume of 50 µL and administered into the right GC muscle. After the second injection, 20 µg antibody was diluted to 25 µL and administered every week for 2 or 6 consecutive weeks. Additionally, only saline was administered to healthy left GC muscle. The antibodies are presented in Table 1.

**Table 1:** Antibody list.

Anibody name	Application	Concentration/Dilution	Company
IGF-I Receptor β	Western blotting /immunostaining	1:1000/1:50	Cell signaling, Danvers, MT, USA
Phospho-IGF-I Receptor β (Tyr1135/1136)	Western blotting	1:1000	Cell signaling
Human/mouse IGF-I Receptor Antibody (Mouse IgG1)	Functional Blocking	20 µg/mice	R&D systems, Minneapolis, MN, USA
Mouse IgG1 Antibody	Control of functional Blocking	20 µg/mice	R&D systems
IGF-I Antibody	Western blotting	1:1000	Abcam, Cambridge, UK
IGF-II Antibody	Western blotting	1:1000	Abcam
Phospho-mTOR (Ser2448) Antibody	Western blotting	1:1000	Cell signaling
Phospho-mTOR (Ser2481) Antibody	Western blotting	1:1000	Cell signaling
mTOR (7C10) Rabbit Monoclonal Antibody	Western blotting	1:1000	Cell signaling
Phospho-FoxO1 (Ser256) Antibody	Western blotting	1:1000	Cell signaling
FoxO1 (C29H4) Rabbit Monoclonal Antibody	Western blotting	1:1000	Cell signaling
Phospho-Akt (Thr308) Antibody	Western blotting	1:1000	Cell signaling
Akt Antibody	Western blotting	1:1000	Cell signaling
Phospho-p70 S6 Kinase (Thr389) (108D2) Rabbit Monoclonal Antibody	Western blotting	1:1000	Cell signaling
p70 S6 Kinase (49D7) Rabbit Monoclonal Antibody	Western blotting	1:1000	Cell signaling
LC3B Antibody	Western blotting	1:1000	Cell signaling
GAPDH Antibody (5A12)	Western blotting	1:10,000	Fujifilm Wako Pure Chemical Co, Osaka, Japan
PAX7 Antibody	Immunostaining	1:50	Developmental Studies Hybridoma Bank(DSHB), Iowa City, IA, USA
Laminin-2 (α-2 Chain) Antibody (4H8-2)	Immunostaining	1:1500	Sigma-Aldrich Japan/Merck, Tokyo, Japan
MyoD Antibody(G-1)	Immunostaining	1:50	Santa Cruz Biotechnology, Dallas, TX, USA
Ki67 Antibody	Immunostaining	1:500	Abcam
Donkey anti-mouse IgG F(ab’)2 (H + L) Alexa Fluor 488	Immunostaining	1:1000	Jackson immunoResearch Laboratories, West Grove, PA, USA
Donkey anti-Rabbit IgG (H + L) Alexa Fluor 647	Immunostaining	1:1000	Jackson immunoResearch Laboratories
Donkey anti-Rat IgG (H + L) Cy3	Immunostaining	1:1000	Jackson immunoResearch Laboratories
Donkey anti-Goat IgG (H + L) Alexa Fluor 647 plus	Immunostaining	1:1000	Molecular probes/Thermo Fisher Scientific, Waltham, MA, USA
Goat anti-mouse IgG (H + L) HRP	Immunostaining	1:5000	Jackson immunoResearch Laboratories
Goat anti-Rabbit IgG (H + L) HRP	Immunostaining	1:5000	Jackson immunoResearch Laboratories
α-Bungarotoxin-Alexa Fluor 594	Immunostaining	1:400	Molecular probes/Thermo Fisher Scientific
Donkey anti-mouse IgG (H + L)	Blocking	1:33 (3%)	Jackson immunoResearch Laboratories

### Footprint test

Footprints were made, and GC muscle function was evaluated by applying black ink to the bottom surface of the hindlimb of the mice and having them walk on a 44 × 10-cm walking path. All results of the footprint tests were blinded, and walking was repeated several times until the best print length and interdigital distance were obtained. Functional assessment was performed on the right (experimental) and left (control) feet using the tibial functional index (TFI) formula, as previously described [33]. Specifically, the distance from the tip of the third toe to the posterior end of the calcaneal process (print length) in both the experimental and control sides was used. The TFI was measured by averaging 10–12 values from a given gait trajectory. A TFI of –100% was defined to indicate complete paralysis and 0% intact muscle function. A footprint test was performed before antibody administration.

### Evaluation of NMJ morphology and side branches

At 2 and 6 weeks after BTX administration, mice were anesthetized, and perfusion fixation was continued transcardially using 4% paraformaldehyde (Sigma-Aldrich Japan/Merck)/phosphate-reduced saline (phosphate-buffered saline [PBS]) at twice the body weight. The GC muscle was then collected, replaced with 30% sucrose/PBS, and embedded in white tissue-coated FL (UI Chemicals Co., Hyogo, Japan).

Sagittal sections (50 µm) were prepared using a cryostat (CM1900)(Leica, Wetzlar, Germany), placed on MAS-coated glass slides (Matsunami Glass, Osaka, Japan), and stored at −20 °C until use. Frozen sagittal sections were washed with PBS for 5 min and then blocked with PBS/10% normal donkey serum (DAKO/Millipore)/0.3% Triton X-100 (PBSDT) for 1 h at room temperature. The anti-IGF-I Receptor β antibody diluted in PBSDT was incubated at 4 °C for 2 days.

Subsequently, the samples were washed thrice for 5 min periods in PBS and incubated with the corresponding secondary antibodies. Alexa Fluor® 594-conjugated α-bungarotoxin protein (NMJ marker) and 4′,6-diamidino-2-phenylindole (DAPI) (nuclei marker) diluted in PBSDT were reacted overnight. Samples were then washed thrice for 5 min periods in PBS and mounted with cover glass using VECTASHIELD Vibrance Antifade Mounting Medium (Vector Laboratories, Burlingame, CA, USA). Observations were made using a BZ-X700 (Keyence Co., Osaka, Japan) or FV-1000D confocal laser microscope (Olympus, Tokyo, Japan).

The NMJ was evaluated using procedures mentioned in previous literature [34]. Briefly, images of the NMJ taken using a confocal laser microscope were analyzed using ImageJ software (https://imagej.net/Welcome). The images were separated into presynaptic and postsynaptic portions and binarized, and the circumference and area were calculated. Twenty items were evaluated (listed in Table 2). Side branches were evaluated using a BZ-X700 (Keyence) with an objective lens at ×20 magnification and a Z-stack pitch of 1 µm. All the experiments were performed in three independent mice.

**Table 2:** Morphological parameter and baseline data.

		Naïve	2 Weeks			6 Weeks		
			cont IgG	IGF1R	*p* value	cont IgG	IGF1R	*p* value
Core variables (pre-synaptic)								
1	Nerve terminal area (µm²)	201.8 (±20.47)	221.1 (±8.063)	218.2 (±1.876)	0.7438	277.9 (±5.774)	220.3 (±23.52)	0.076
2	Nerve terminal perimeter (µm)	287.7 (±19.92)	237.2 (±12.66)	224.4 (±0.9493)	0.3691	314.8 (±14.47)	255.7 (±12.88)	0.0379*
3	Number of terminal branches	27.07 (±2.821)	18.84 (±2.894)	16.04 (±0.0869)	0.3873	39.55 (±5.636)	29.83 (±5.552)	0.2863
4	Number of branch points	22.03 (±3.382)	27.5 (±2.363)	24.2 (±0.5035)	0.2437	36.33 (±1.832)	28.93 (±4.110)	0.1754
5	Total length branches (µm)	136.6 (±11.17)	134.3 (±7.489)	124.9 (±0.2517)	0.2765	168.1 (±4.548)	135.2 (±9.592)	0.0361*
Core Variables (post-synaptic)								
6	AChR area (µm²)	291 (±17.13)	264.6 (±16.94)	240.7 (±11.28)	0.3054	268.7 (±2.040)	217.3 (±17.99)	0.047*
7	AChR perimeter (µm)	285.4 (±18.72)	193 (±6.205)	192.7 (±1.605)	0.961	326.2 (±12.43)	273.9 (±15.65)	0.0592
8	Endplate area (µm²)	527.2 (±26.82)	364.3 (±23.30)	342.7 (±11.02)	0.4485	484.5 (±15.14)	383.8 (±32.97)	0.05
9	Endplate perimeter (µm)	103.5 (±3.043)	97.37 (±4.206)	91.89 (±0.8911)	0.2714	118.3 (±3.617)	97.72 (±2.194)	0.0083**
10	Endplate diameter (µm)	36.1 (±1.330)	39.41 (±2.756)	36.35 (±0.2046)	0.3299	46.47 (±0.9575)	38.44 (±0.1193)	0.0011**
11	Number AChR clusters	3.017 (±0.7303)	1.992 (±0.1949)	2.25 (±0.1665)	0.3705	7.367 (±0.4693)	5.775 (±0.9468)	0.2065
Derived variables (pre-synaptic)								
12	Average length branches (µm)	5.797 (±0.1821)	8.482 (±0.9082)	8.969 (±0.1469)	0.6248	5.225 (±0.5845)	5.422 (±0.9590)	0.8695
13	Complexity	4.795 (±0.1527)	4.699 (±0.1148)	4.525 (±0.0135)	0.2066	5.193 (±0.0848)	4.852 (±0.1316)	0.0955
Derived variables (post-synaptic)								
14	Average area AChR clusters (µm²)	152.5 (±39.05)	184.1 (±16.13)	151.8 (±15.86)	0.2257	68.73 (±4.306)	60.93 (±9.695)	0.5029
15	Fragmentation	0.4797 (±0.1008)	0.2894 (±0.0668)	0.3615 (±0.0504)	0.4378	0.7102 (±0.0254)	0.67 (±0.0488)	0.5055
16	Compactness (%)	56.22 (±0.3155)	73.52 (±0.1686)	70.74 (±1.120)	0.0706	56.78 (±1.969)	57.71 (±1.024)	0.6956
17	Overlap (%)	63.1 (±5.104)	74.84 (±2.246)	78.99 (±0.7334)	0.1541	73.59 (±1.596)	73.2 (±3.398)	0.9229
18	Area of synaptic contact (µm²)	182.1 (±17.02)	195.6 (±6.963)	188.1 (±6.126)	0.4641	198.4 (±5.746)	161.5 (±17.15)	0.1107
Associated nerve and muscle variables								
19	Axon diameter (µm)	1.579 (±0.2782)	1.971 (±0.0665)	2.126 (±0.0773)	0.2031	2.128 (±0.2366)	1.856 (±0.1489)	0.3852
20	Number of axonal inputs	1.175 (±0.0804)	1.633 (±0.0682)	1.85 (±0.1750)	0.3129	1.925 (±0.0661)	1.592 (±0.1294)	0.0835

Twenty morphological parameters are included in this examination analyzed with the images shown in Fig. 2F. Control IgG treatment is represented as cont IgG and anti-insulin-like growth factor 1 receptor (IGF1R) antibody treatment as IGF1R. *p < 0.05, **p < 0.01, analyzed with one-way ANOVA followed by post-hoc Tukey’s test.

*AChR* acetylcholine receptors.

### Immunohistochemical staining of muscle tissue

At 2 and 6 weeks after BTX administration, mice were anesthetized with an anesthetic mixture of three reagents and euthanized by exsanguination. The GC muscle was collected without fixation and its weight was measured. The GC muscle was subsequently flash frozen in liquid nitrogen and embedded in a white tissue-coated FL. Horizontal sections (20 µm) were prepared using a cryostat and placed on MAS-coated glass slides. The samples were stored at −80 °C until use. Horizontal frozen sections were immersion fixed in 4% paraformaldehyde for 3 min.

After washing with PBS, the samples were immersed in 0.01 M citric acid solution (pH 6.0) and boiled for antigen retrieval. The samples were cooled under running water for 30 min to room temperature. After washing with PBS, blocking was performed for 1 h in PBSDT. Subsequently, blocking treatment was performed on endogenous IgG by reacting with 3% donkey anti-mouse IgG/PBS solution for 1 h. The samples were washed thrice for 5 min periods in PBS and incubated with diluted anti-IGF-I Receptor β antibody, anti-Pax7 antibody (SC marker), anti-laminin antibody (muscular membrane marker), anti-Ki67 (cell proliferation marker), or anti-myoblast determination protein 1 (MyoD) (myoblast marker)antibodies at 4 °C for 2 days.

The samples were subsequently washed thrice with PBS for 5 min periods and reacted with a mixture of the corresponding secondary antibody and DAPI diluted in PBS at 4 °C overnight. All the slides were treated with the Vector TrueVIEW Autofluorescence Quenching Kit (Vector Laboratories) for 1 min to reduce autofluorescence and then sealed. Observations were made using a BZ-X700 (Keyence) or FV-1000D confocal laser microscope (Olympus).

### Measurement of the cross-sectional area of the muscle and number of Pax7^+^-SCs

Horizontal sections obtained below the center of the muscle belly were prepared to quantify the number of muscle fibers and to measure the cross-sectional area. Immunohistochemical staining with an anti-laminin antibody was performed. The cross-sectional area of the muscle fibers was subsequently analyzed using BZ-H3C Hybrid Cell Count Software (Keyence). Thereafter, triplicate staining was performed with anti-laminin and anti-Pax7 antibodies and DAPI. Four horizontal images of the medial and lateral heads of the GC muscle were acquired (a total of eight images) using a 20× objective lens to measure the number of Pax7^+^-SCs per mouse.

The number of myofibers was measured in the area surrounded by laminin, and Pax7^+^-SCs were visually assessed for DAPI and Pax7 double positive cells. The ratio of Pax7^+^-SCs per muscle fiber was calculated from the number of muscle fibers measured and the number of Pax7^+^-SCs. Triple staining was performed with anti-laminin, anti-Pax7, and anti-Ki67 antibodies, and the same procedure was used to measure the number of Ki67^+^ Pax7^+^-SCs. Triple staining was performed with anti-laminin and anti-myoblast determination protein 1 (MyoD) antibodies and DAPI, and the same procedure was used to measure the number of DAPI and MyoD double positive cells. All the measurements were performed in a blinded manner. All the experiments were performed in four independent mice.

### Immunoblotting

GC muscle homogenates were prepared from four independent mice. The GC muscle was homogenized in a radioimmunoprecipitation assay buffer (WAKO) containing Protease Inhibitor Cocktail Set III (Fujifilm Wako Pure Chemical Co, Osaka, Japan), PhosSTOP (Roche Diagnostics, Mannheim, Germany), and 2 mM ethylene glycol tetraacetic acid (Fujifilm Wako Pure Chemical Co.). Protein concentrations were determined using a bicinchoninic acid assay (Thermo Fisher Scientific, Waltham, MA, USA).

Sample preparation and western blotting were performed as described previously [35, 36], with minor modifications. Briefly, homogenates of the GC muscle were separated on either 10.5% or 18% sodium dodecyl sulfate-polycrylamide gel electrophoresis gels and electrically transferred to polyvinylidene fluoride membranes (Fujifilm Wako Pure Chemical Co.). The protein-transferred membranes were incubated for 20 min in Blocking One (Nakarai, Kyoto, Japan). After washing with 50 mM Tris-HCl (pH 7.4), 150 mM NaCl, and 0.05% Tween 20 (T-TBS), the membranes were incubated overnight with primary antibodies diluted in 5% Blocking One/T-TBS buffer.

After washing thrice in T-TBS, the membranes were incubated for 1 h with horseradish peroxidase-conjugated secondary antibodies in 5% Blocking One/T-TBS buffer. Thereafter, immunoreactivities were detected using the ECL system (GE Healthcare, Tokyo, Japan). Chemiluminescence was captured using ImageQuant 800 (AMERSHAM/GE Healthcare Japan, Tokyo, Japan). Band intensities were measured using ImageJ software for quantification. Full images of western blots are presented in Supplementary Fig. S1. The antibodies are presented in Table 1.

### RNA extraction and quantitative reverse transcription-polymerase chain reaction (qRT-PCR)

RNA extraction and qRT-PCR were performed as previously described, with some modifications [35]. Briefly, the GC muscle was dissected and flash frozen in liquid nitrogen. The frozen samples were then ground into powder using BioMasher II (Nippi, Tokyo, Japan), and total RNA was extracted using Isogen II (Nippon Gene, Tokyo, Japan). First-strand cDNA was synthesized using the ReverTra Ace qPCR RT Master Mix with gDNA Remover (Toyobo, Osaka, Japan).

qRT-PCR was subsequently performed using the THUNDERBIRD SYBR qPCR Mix (Toyobo) and Thermal Cycler Dice Real Time System II TP900 (Takara Bio, Shiga, Japan). The relative copy number of various gene transcripts per β-actin transcript was determined using calibration standards for each of the tested molecules. GC muscle was harvested from three independent mice. The primers are listed in Table 3.

**Table 3:** Primer list.

Gene name	Sequence	
*Actb (actin, beta)*	Forward	TTTGCAGCTCCTTCGTTGC
	Reverse	ACGATGGAGGGGAATACAGC
*Lrp4 (low density lipoprotein receptor-related protein 4)*	Forward	GTGTGGCAGAACCTTGACAGTC
	Reverse	TACGGTCTGAGCCATCCATTCC
*Hdac4 (histone deacetylase 4)*	Forward	AGCAGGAGCTGCTCTTCAGACA
	Reverse	ACAGAGGTCTGTGGCTGCCAAA
*Ache (acetylcholinesterase)*	Forward	TTCCTTCGTGCCTGTGGTAGAC
	Reverse	CCGTAAACCAGAAAGTAGGAGCC
*Chrna1 (cholinergic receptor, nicotinic, alpha polypeptide 1)*	Forward	CTCTCGACTGTTCTCCTGCTG
	Reverse	GTAGACCCACGGTGACTTGTA
*Chrnd (cholinergic receptor, nicotinic, delta polypeptide)*	Forward	TGAGAAGGGCTACGACAAAGAC
	Reverse	GTCTCCTCCACTTCTTTCAGGG
*Chrne (cholinergic receptor, nicotinic, epsilon polypeptide)*	Forward	GAAGCCACTGGAGAGGAACTG
	Reverse	AGGGAGATCAGGAACTTGGTTG
*Musk (muscle, skeletal, receptor tyrosine kinase)*	Forward	GCTGGAAGTGGAGGAAGACAG
	Reverse	GTGCAGCGTAGGGTTACAAAG
*Myog (myogenin)*	Forward	ATCTCCGCTACAGAGGCGGG
	Reverse	TAGGGTCAGCCGCGAGCAAA
*Fbxo32/MAFbx/atrogin-1 (F-box protein 32)*	Forward	CAACATTAACATGTGGGTGTAT
	Reverse	GTCACTCAGCCTCTGCAT
*Trim63/MuRF1 (tripartite motif-containing 63)*	Forward	GAGAACCTGGAGAAGCAGCT
	Reverse	CCGCGGTTGGTCCAGTAG

### Statistical analyses

Unpaired *t*-tests for two-group comparisons, the one-way analysis of variance (ANOVA) method, Tukey’s multiple tests for comparisons among three or more groups, and the two-way ANOVA method and Tukey’s multiple tests for the comparisons of progress were used. Data were presented as the mean ± standard errors. All statistical analyses were performed using Prism software (GraphPad Software, Boston, MA, USA). Statistical comparisons of measurements were considered significant at a 95% confidence level (*P* < 0.05).

## Results

### Anti-IGF1R antibody administration prolongs the effect of BTX

**Fig. 1:**
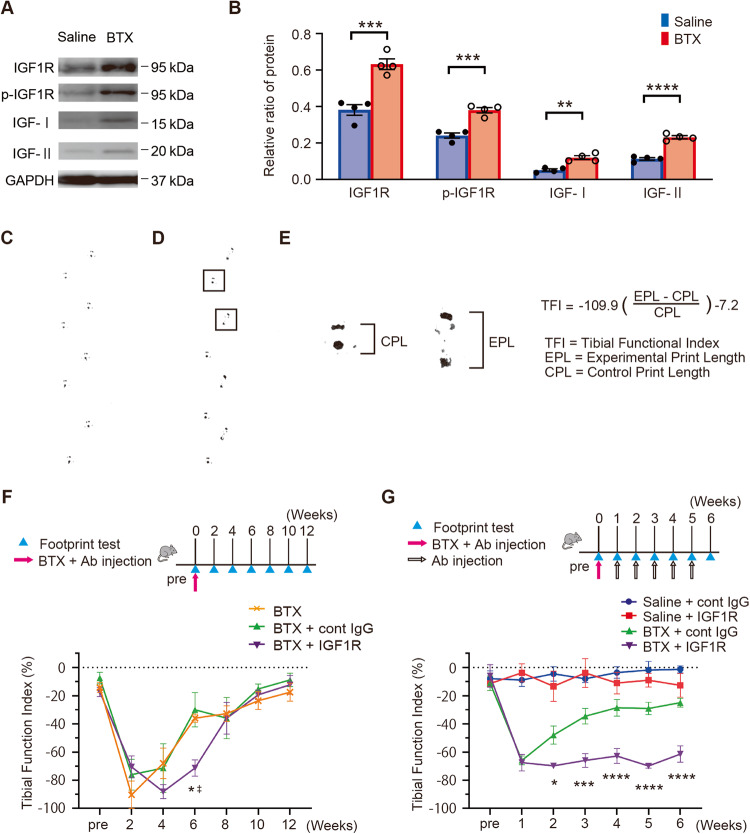
The paralysis duration of the gastrocnemius (GC) muscles induced by botulinum toxin-A (BTX) is extended by anti-insulin-like growth factor 1 receptor (IGF1R) antibody. **A** Representative images of western blot analysis of IGF signaling pathway in mouse GC muscle 2 weeks after saline or BTX injection. Glyceraldehyde-3-phosphate dehydrogenase (GAPDH) is used as a loading control. **B** Quantification of IGF1R, phosphorylated IGF1R, IGF-I, and IGF-II protein levels in comparison to GAPDH protein levels. Values are presented as the mean ± standard error of the mean (SEM). **P < 0.01, ***P < 0.001, and ****P < 0.0001, analyzed using two-tailed unpaired Student’s *t-*test (*N* = 4 per group). Representative footprint analysis of naïve (**C**) or BTX- injected (**D**) mice. **E** Enlarged view of footprints shown in **D** and the formula for calculating the tibial functional index (TFI) (−100% indicates complete paralysis, and 0% indicates an intact muscle) are shown. Time schedule of the footprint test and single (**F**) or sequential (**G**) antibody administration (20 µg) after saline or BTX (F, 2.5 U; G, 1.25 U) treatment at the beginning. Control IgG antibody treatment is represented as cont IgG and anti-IGF1R antibody treatment as IGF1R. Values are presented as the mean ± SEM. *P < 0.05, ***P < 0.001, ****P < 0.0001, and ^‡^*P* < 0.01, analyzed using two-way analysis of variance followed by post-hoc Tukey’s test (*N* = 3 or 4 per group).

First, western blot analyses were performed to examine the protein levels of IGF-I, IGF-II, IGF1R, and phosphorylated-IGF1R in the GC muscle 2 weeks following BTX treatment. As shown in Fig. 1A, B and Supplementary Fig. S1A, all the protein levels compared to glyceraldehyde-3-phosphate dehydrogenase (GAPDH) increased after BTX administration. The average IGF1R/GAPDH ratio before and after BTX administration was 0.38 ± 0.029 (*N* = 4) and 0.63 ± 0.029 (*N* = 4), respectively (*P* = 0.0009, two-tailed unpaired Student’s *t-*test); the average p-IGF1R/GAPDH ratio after saline and BTX administration was 0.24 ± 0.014 (*N* = 4) and 0.38 ± 0.015 (*N* = 4), respectively (*P* = 0.0005, two-tailed unpaired Student’s *t-*test). Further, the average IGF-I/GAPDH ratio after saline and BTX administration was 0.052 ± 0.0065 (*N* = 4) and 0.12 ± 0.011 (*N* = 4), respectively (*P* = 0.0016, two-tailed unpaired Student’s *t-*test). Moreover, the average IGF-II/GAPDH ratio after saline and BTX administration was 0.11 ± 0.0071 (*N* = 4) and 0.23 ± 0.0085 (*N* = 4), respectively (P < 0.0001, two-tailed unpaired Student’s *t-*test).

The results of western blotting against the p-IGF1R/ IGF1R ratio are shown in Supplementary Figures S1C and S2A. IGF1R antibody treatment reduced the p-IGF1R/IGF1R ratio in the GC muscle by 40%.

The TFI values are shown in Fig. 1C–E. The treatment and test schedule are shown in Fig. 1F and G, respectively. As shown in Fig. 1F, 2.5 U BTX treatment in the GC muscle induced nearly complete paralysis at 2 or 4 weeks, followed by a spontaneous recovery until 12 weeks. Paralysis in the GC muscle at 6 weeks following treatment was significantly longer by 2 weeks in the anti-IGF1R antibody group than in the BTX alone and control IgG (cont IgG) groups.

With respect to the effects of sequential injections of anti-IGF1R or mouse cont IgG, with or without 1.25 U BTX administration, the TFI of the anti-IGF1R antibody alone group was not significantly different from that of the cont IgG group. In contrast, at 6 weeks after BTX injection, the TFI of the anti-IGF1R antibody group remained low, whereas that of the cont IgG-treated group spontaneously recovered (Fig. 1G). These results indicated that BTX-induced paralysis was significantly sustained by the anti-IGF1R antibody. However, there was no decrease in the TFI when anti-IGF1R antibody or cont IgG alone was administered, supporting that BTX treatment induced the expression of IGF-I, IGF-II, and IGF1R in the GC muscle and that these signals contributed to the spontaneous recovery from BTX treatment.

### The NMJ**, but** not sprouting fibers, is regulated by the IGF signaling pathway

**Fig. 2:**
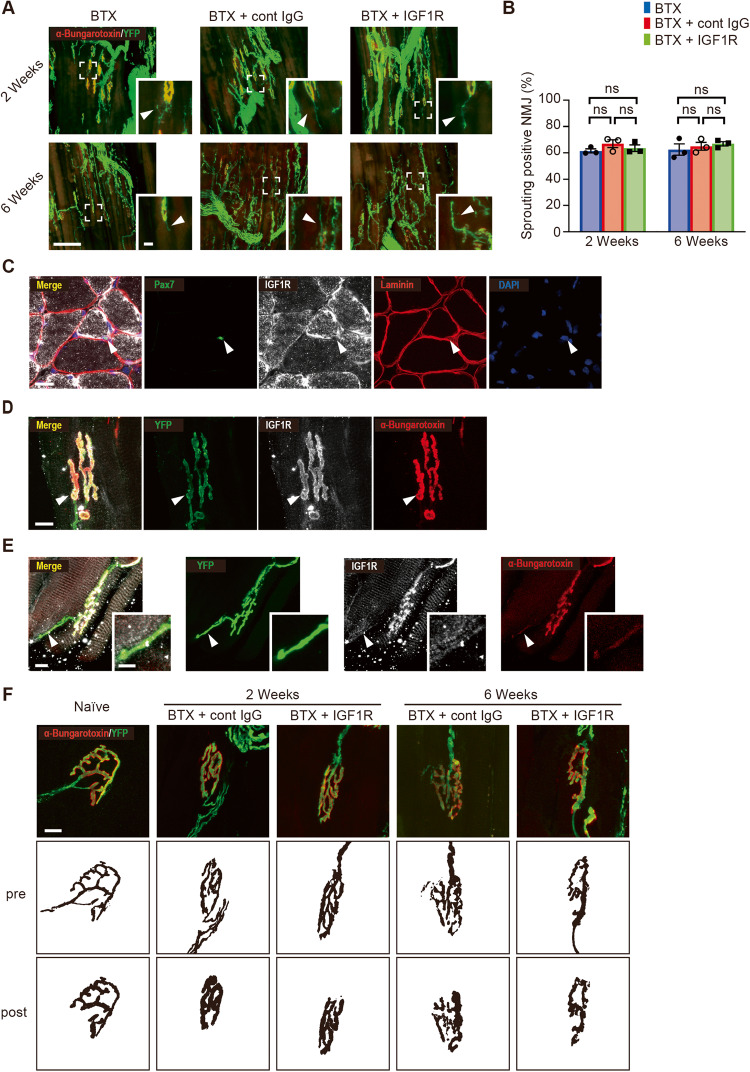
Morphological analysis of the sprouting fibers and neuromuscular junction (NMJ). **A** Confocal images with Thy1-YFP (green) and α-bungarotoxin (red) at 2 (upper) and 6 (lower) weeks after botulinum toxin-A (BTX) administration. Control IgG treatment is represented as cont IgG and anti-insulin-like growth factor 1 receptor (IGF1R) antibody treatment as IGF1R. Scale bar = 100 µm. The right panel indicates the enlarged view of the white-square–labeled area in the left panel. Arrowheads indicate sprouting fibers. Scale bar = 10 µm. **B** Percentage of the NMJ with sprouting fibers. Data are presented as the mean ± standard error of the mean. ns, not significant, analyzed with one-way analysis of variance followed by post-hoc Tukey’s test (*N* = 3). **C** Immunohistochemical images of Pax7 (green), IGF1R (white), laminin (red), and 4′,6-diamidino-2-phenylindole (DAPI) (blue) in the gastrocnemius (GC) muscle of wild-type mice. Arrowheads indicate Pax7^+^-satellite cells (SCs). Scale bar = 40 µm. **D** Confocal images of YFP (green), IGF1R (white), and α-bungarotoxin (red) in the NMJ of Thy1-YFP mice. Scale bar = 10 µm. Arrowheads indicate the NMJ. **E** Confocal images of YFP (green), IGF1R (white), and α-bungarotoxin (red) in the sprouting fibers of Thy1-YFP mice. Scale bar = 10 µm. The right panel indicates the enlarged view of the white-square–labeled area in left panel. Arrowheads indicate sprouting fiber. **F** Immunohistochemical analysis of YFP (presynaptic components, green) and α-bungarotoxin (postsynaptic components, red) at 2 or 6 weeks after BTX and antibody administration into the GC muscle. Presynaptic axon terminals are represented as pre and postsynaptic acetylcholine receptor clusters as post. Scale bar = 10 µm.

BTX treatment induced multiple sprouting fibers from the proximal position of GC-innervated nerve at 2 and 6 weeks (Fig. 2A). However, there was no significant difference in the percentage of sprouting fibers from the proximal main fibers between the cont IgG and anti-IGF1R antibody groups (Fig. 2B). Immunohistochemical analysis of the GC muscle and NMJ showed that IGF1R was highly expressed in the membrane of muscle fibers, Pax7^+^-SCs (Fig. 2C), and NMJ (Fig. 2D). However, it was weakly expressed in the growing tip of the sprouting fiber (Fig. 2E). Particularly, IGF1R expression was completely co-localized with fluorescent dye-conjugated α-bungarotoxin staining (nicotinic acetylcholine receptors), indicating that IGF1R existed mainly on the postsynaptic membrane. These results suggested that IGF1R may act on the muscle fibers, postsynapse of the NMJ, or Pax7^+^-SCs, rather than on the sprouting fibers.

Thereafter, the morphological changes in the NMJ following BTX and anti-IGF1R antibody or cont IgG treatment are shown in Fig. 2F and Table 2. Presynaptic and postsynaptic components were significantly lower in BTX-treated samples than in naïve controls 2 weeks after treatment. Particularly, BTX treatment significantly reduced postsynaptic components in the cont IgG and anti-IGF1R antibody groups.

At 6 weeks after BTX treatment and antibody administration, presynaptic and postsynaptic components largely recovered in the cont IgG group (Fig. 2F and Table 2). Compared with the cont IgG group, the anti-IGF1R antibody group demonstrated a lack of regeneration of both presynaptic and postsynaptic components (e.g., acetylcholine receptors area, endplate perimeter, and endplate diameter). We also confirmed our results concerning the number of sprouting fibers. The number of axonal inputs to the NMJ was not significantly different between the cont IgG and anti-IGF1R antibody groups. However, BTX treatment itself increased axonal sprouting fibers (Fig. 2A, B). These results suggested that the NMJ, but not sprouting fibers, was regulated by the IGF1R signaling pathway.

### IGF1R signaling pathway regulates recovery from neurogenic atrophy by BTX

**Fig. 3:**
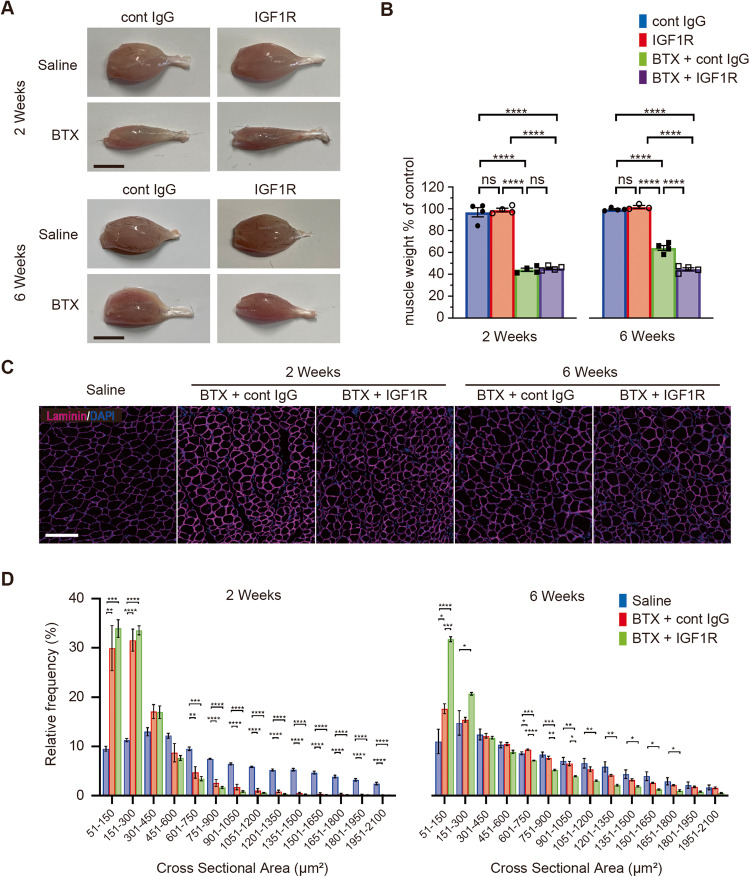
Spontaneous recovery of neurogenic atrophy in the gastrocnemius (GC) muscles is inhibited by anti-insulin-like growth factor 1 receptor (IGF1R) antibody administration. **A** Representative images of the GC muscle. Scale bar = 5 mm. **B** The muscle wet weight as a percentage of contralateral control muscle at 2 weeks and 6 weeks after botulinum toxin-A (BTX) and antibody-treatment. Control IgG antibody treatment is represented as cont IgG and anti-IGF1R antibody treatment as IGF1R. Data are presented as the mean ± standard error of the mean (SEM). ns, not significant, ****P < 0.0001 analyzed with one-way analysis of variance (ANOVA) followed by post-hoc Tukey’s test (*N* = 3 or 4 per group). **C** Double immunostaining images of laminin (magenta) and 4′,6-diamidino-2-phenylindole (DAPI) (blue) in the horizontal section of the GC muscle. Scale bar = 100 µm. **D** Histograms of the distribution of GC muscle fiber size at 2 or 6 weeks after treatment. Values are presented as the mean ± SEM. *P < 0.05, **P < 0.01, ***P < 0.001, ****P < 0.0001, analyzed using one-way ANOVA followed by post-hoc Tukey’s test (*N* = 4 per group).

Given that IGF1R was expressed in the plasma membrane of the muscle fibers, the sequential effect of the administration of anti-IGF1R antibody on the muscle fibers was analyzed. The weight of the denervated GC muscle was significantly lower by approximately 40% than that of the contralateral control GC muscle at 2 weeks following BTX administration (Fig. 3A, B).

Meanwhile, the muscle appearance or wet weight percentage of either antibody group was not significantly different from that of the cont IgG group (cont IgG: 96.8 ± 4.3% [*N* = 4] vs. IGF1R: 98.9 ± 1.6% [*N* = 4] vs. BTX+cont IgG: 44.2 ± 1.6% [*N* = 4] vs. BTX + IGF1R: 45.6 ± 1.0% [*N* = 4], *P* < 0.0001, one-way ANOVA). However, at 6 weeks after treatment, muscle weight in the cont IgG group was spontaneously recovered to 64.2 ± 2.3% of the contralateral control muscle weight. In contrast, the anti-IGF1R antibody group did not show any recovery in the muscle weight and maintained 44.7 ± 1.5% of the contralateral muscle weight (cont IgG: 99.5 ± 0.7% [*N* = 4] vs. IGF1R: 102 ± 1.3% [*N* = 3] vs. BTX + cont IgG: 64.2 ± 2.3% [*N* = 4] vs. BTX + IGF1R: 44.7 ± 1.5% [*N* = 4], *P* < 0.0001, one-way ANOVA).

We subsequently performed a histological analysis of the cross-sectional area of the muscle fibers (Fig. 3C). The frequency distribution of the cross-sectional area of the muscle fibers is shown in Fig. 3D. At 2 weeks after BTX and antibody administration, the numbers of muscle fibers with small cross-sectional areas, such as those of 51–150 or 151–300 µm^2^, were significantly higher in both antibody-treated groups than in the saline-treated group. Compared with the saline-treated group, the BTX-treated group had fewer muscle fibers with a larger cross-sectional area. These results represented neurogenic atrophy induced by BTX. The frequency of muscle fibers in smaller cross-sectional areas was significantly higher in the BTX-treated group than in the saline-treated group; however, it was not different between the cont IgG and anti-IGF1R antibody groups.

In contrast, the distribution of muscle fibers in the anti-IGF1R antibody group was significantly different from that in the cont IgG group at 6 weeks. The distribution was spontaneously recovered after 6 weeks in the cont IgG group, while the anti-IGF1R antibody group showed a higher frequency of small muscle fibers with cross-sectional areas of 51–150 and 151–300 µm^2^ and a lower frequency of muscle fibers with cross-sectional areas of >451 µm^2^. These results suggested that BTX treatment induced neurogenic atrophy and that sequential treatment with anti-IGF1R antibody maintained the neurogenic atrophy induced by BTX.

### Sequential administration of anti-IGF1R antibody following BTX administration increases the number of Pax7^+^-SCs in the GC muscle

**Fig. 4:**
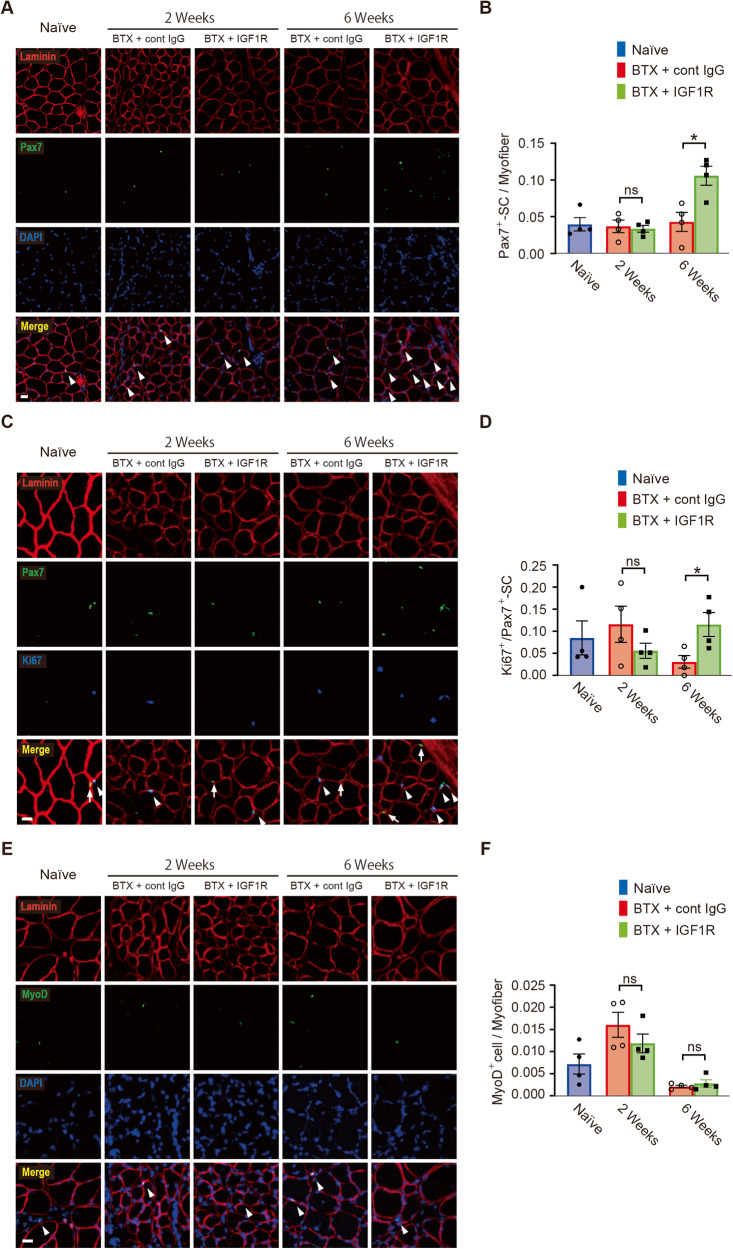
Anti-insulin-like growth factor 1 receptor (IGF1R) antibody administration increases the number of Pax7^+^-satellite cells (SCs) in the gastrocnemius (GC) muscle treated with botulinum toxin-A (BTX). **A** Representative cross-sectional images of the GC muscle stained with anti-Pax7 (green), 4′,6-diamidino-2-phenylindole (DAPI) (blue), and anti-laminin (red). Control IgG antibody treatment is represented as cont IgG and anti-IGF1R antibody treatment as IGF1R. White arrowheads indicate Pax7^+^-SCs. Scale bar = 20 µm. **B** Quantification of the number of Pax7^+^-SCs per myofiber at 2 or 6 weeks after BTX and antibody administration. Values are presented as the mean ± standard error of the mean (SEM). ns, not significant, *P < 0.05, analyzed with two-tailed unpaired Student’s *t*-test (*N* = 4 per group). **C** Representative cross-sectional images of the GC muscle stained with anti-Pax7 (green), anti-Ki67 (blue), and anti-laminin (red). White arrows indicate Ki67^-^ Pax7^+^-SCs; white arrowheads indicate Ki67^+^ Pax7^+^-SC; scale bar = 20 µm. **D** Quantification of the number of Ki67^+^ Pax7^+^-SCs per myofiber at 2 or 6 weeks after BTX and antibody administration. Values are presented as the mean ± SEM. ns not significant, *P < 0.05, analyzed with two-tailed unpaired Student’s *t-*test (*N* = 4 per group). **E** Representative cross-sectional images of the GC muscle stained with anti-MyoD (green), DAPI (blue), and anti-laminin (red). White arrowheads indicate MyoD^+^ cells. Scale bar = 20 µm. **F** Quantification of MyoD^+^ cells per myofiber at 2 or 6 weeks after BTX and antibody administration. Values are presented as the mean ± SEM. ns, not significant, analyzed with two-tailed unpaired Student’s *t-*test (*N* = 4 per group).

Given that IGF1R was expressed in Pax7^+^-SCs, we hypothesized that administering anti-IGF1R antibody following BTX treatment would affect the repopulation and differentiation of SC and NMJ regeneration by BTX treatment. Thus, immunohistochemical analysis for Pax7 was performed and the ratio of SCs per muscle fiber was compared among the naïve control, BTX+cont IgG, and BTX+anti-IGF1R antibody groups (Fig. 4A). The ratio of SCs in the GC muscle was not significantly different between two groups at 2 weeks after treatment (BTX+cont IgG: 0.037 ± 0.0084 [*N* = 4] vs. BTX + IGF1R: 0.034 ± 0.0046 [*N* = 4], *P* = 0.75, unpaired Student’s *t-*test) (naïve: 0.040 ± 0.0090 [*N* = 4]) (Fig. 4A, B). However, the ratio of SCs was significantly higher in the BTX+anti-IGF1R antibody group than in the BTX+cont IgG group 6 weeks after treatment (BTX+cont IgG: 0.043 ± 0.013 [*N* = 4] vs. BTX + IGF1R: 0.11 ± 0.013 [*N* = 4], *P* = 0.014, unpaired Student’s *t-*test).

To examine the effect on Pax7^+^-SC proliferation, immunohistochemical staining for Ki67 was performed (Fig. 4C). The ratio of Ki67^+^ cells per Pax7^+^-SC in the GC muscle in the BTX+cont IgG and BTX + IGF1R groups was not significantly different from that in the naïve control group at 2 weeks after treatment (BTX+cont IgG: 0.12 ± 0.041 [*N* = 4] vs. BTX + IGF1R: 0.056 ± 0.017 [*N* = 4], *P* = 0.23, unpaired Student’s *t-*test) (naïve: 0.085 ± 0.038 [*N* = 4]) (Fig. 4C, D). However, the ratio of Ki67^+^ cells per Pax7^+^-SC in the BTX + anti-IGF1R antibody group was significantly higher than that in the BTX + cont IgG group 6 weeks after treatment (BTX + cont IgG: 0.031 ± 0.014% [*N* = 4] vs. BTX + IGF1R: 0.12 ± 0.027% [*N* = 4], *P* = 0.033, unpaired Student’s *t-*test).

Immunohistochemistry for MyoD was performed to examine its effect on SC differentiation (Fig. 4E). There was no significant change in the ratio of MyoD^+^ cells per muscle fiber at both 2 weeks after treatment (BTX + cont IgG: 0.016 ± 0.0028% [*N* = 4] vs. BTX + IGF1R: 0.012 ± 0.0021% [*N* = 4], *P* = 0.28, unpaired Student’s *t-*test) (naïve: 0.0072 ± 0.0023% [*N* = 4]) and 6 weeks after treatment (BTX + cont IgG: 0.0021 ± 0.0003% [*N* = 4] vs. BTX + IGF1R: 0.0028 ± 0.00084% [*N* = 4], *P* = 0.47, unpaired Student’s *t-*test) (Fig. 4E, F).

### IGF1R inhibition regulates the NMJ via the mTOR/S6 kinase

**Fig. 5:**
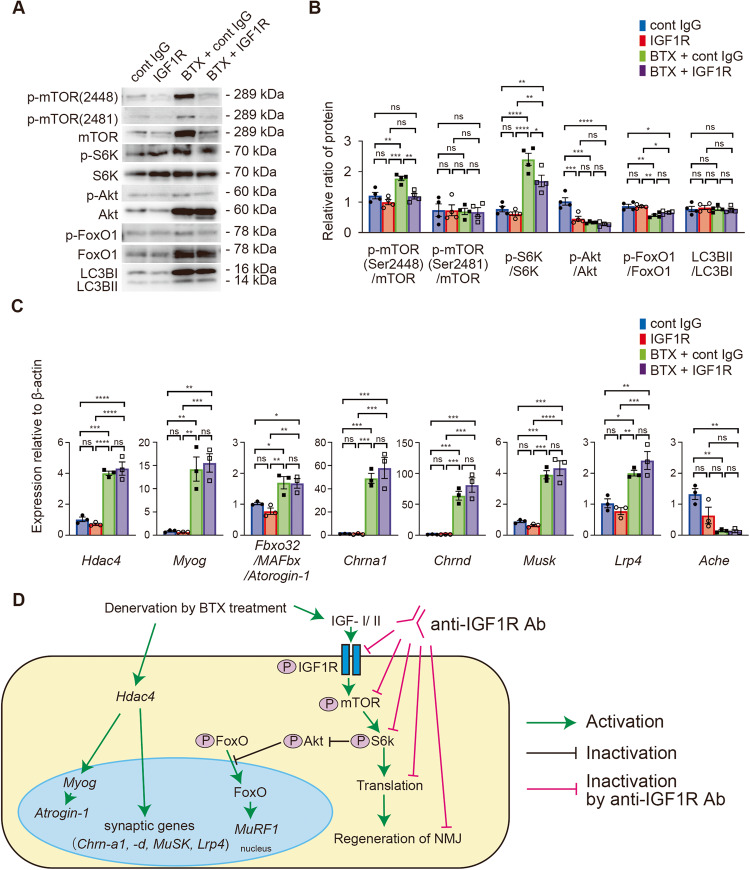
Anti-insulin-like growth factor 1 receptor (IGF1R) antibody administration inhibits specific signaling molecules involved in the regulation of the neuromuscular junction (NMJ). **A** Representative images of western blot analysis of downstream signaling molecules of IGF1R in mouse gastrocnemius (GC) muscles at 2 weeks after saline or botulinum toxin-A (BTX) injection with antibody administration. Control IgG antibody treatment is represented as cont IgG and anti-IGF1R antibody treatment as IGF1R. **B** Quantification of the p-mTOR (S2448)/mTOR, p-mTOR (S2481)/mTOR, p-S6K/S6K, p-Akt/Akt, p-FoxO1/FoxO1, and light chain 3BII/light chain 3BI(LC3BII/LC3BI) ratios. Values are presented as the mean ± standard error of the mean (SEM), ns, not significant, *P < 0.05, **P < 0.01, ***P < 0.001, ****P < 0.0001, analyzed with one-way ANOVA followed by post-hoc Tukey’s test (*N* = 4 per group). **C** Transcript levels of *Hdac4*, *Myog*, *Fbxo32*, *Chrna1*, *Chrnd*, *Musk*, *Lrp4*, and *Ache* in the GC muscle 2 weeks following saline or BTX injection with an antibody treatment. Values are presented as mean ± SEM, ns, not significant, *P < 0.05, **P < 0.01, ***P < 0.001, ****P < 0.0001, analyzed with one-way ANOVA followed by post-hoc Tukey’s test (*N* = 3 per group). **D** Scheme illustrating the role of IGF signaling pathway in the muscle response to denervation by BTX and signaling pathway affected by anti-IGF1R antibody administration.

Denervation by BTX strongly activated the expression of total mTOR and Akt without inducing autophagy (Fig. 5A), which was detected according to the light chain 3BII/light chain 3BI (LC3BII/LC3BI) ratio. In addition, phosphorylated mTOR (Ser2448) and S6 kinase were more significantly activated than total mTOR and S6 kinase (Fig. 5B). On qRT-PCR (Fig. 5C), all genes, except *Trim63/MuRF1* and *Chrne*, were upregulated by BTX treatment (data not shown), although none of them were affected by anti-IGF1R antibody treatment. However, the relative expression of phosphorylated mTOR and S6 kinase was significantly affected by anti-IGF1R antibody administration.

Interestingly, the total protein levels of mTOR, S6 kinase, and forkhead box protein (Fox) O1 were reduced by anti-IGF1R antibody treatment. These results suggested that BTX treatment activated various signaling pathways; however, inhibition of IGF1R signaling mainly blocked the mTOR/S6 kinase pathway for the translation signals and NMJ regeneration (Fig. 5D).

## Discussion

This study found that BTX treatment of the GC muscle upregulated the protein levels of IGF1R, and anti-IGF1R antibody administration prolonged the effects of BTX. Additionally, the NMJ, but not sprouting fibers, was regulated by the IGF1R signaling pathway in the GC muscle. BTX-treated muscles spontaneously recovered from neurogenic atrophy, which was suppressed by IGF1R inhibition. Sequential administration of anti-IGF1R antibody increased the number of Pax7^+^-SCs in the GC muscle, but only at 6 weeks after treatment. Consistent with other nerve injury models [37, 38], BTX treatment strongly upregulated the mTOR/S6 kinase signaling pathway or transcription of synaptic components, but not autophagy induction, confirmed by LC3BII/LC3BI ratio [39]. Finally, IGF1R inhibition affected only mTOR/S6 kinase translational signaling pathway in the GC muscle. These results suggested that IGF1R inhibition suppresses mTOR/S6 kinase and NMJ regeneration.

With respect to IGF-I, IGF-II, and IGF1R expression in the muscle injected with BTX, previous reports suggested that the IGF1R signaling pathway is involved in the recovery from BTX-induced denervation in rat GC [40] and rabbit eyelid [41] muscles. Additionally, one study demonstrated that IGF-I expression in the rat gluteus muscle was gradually increased in the myocytes from 12 h after BTX administration [42], peaking at day 2. IGF-II expression reportedly increases 8 days after administration. This upregulation of the IGF-I and IGF-II was confirmed in this study, and positive IGF1R expression in the muscles, SCs, and NMJ was newly revealed on immunohistochemical analysis. These results provide a rationale for focusing on the IGF1R signaling pathway in the research of NMJ.

Although IGF1R suppression is evident to prolong muscle paralysis, it was still unclear which cell was the target of the anti-IGF1R antibody. To the best of our knowledge, live imaging experiments have demonstrated the repair process of BTX-induced paralyzed endplates [8]. Within a few days, the nerve terminal loses its function with an increased number of newly sprouting fibers. After 28 days, only the additional sprouting fibers are functional, and the parent terminals are not recovered at the time. However, when the original parent terminals are recovered, the sprouting fibers are eliminated in a later phase [8]. We hypothesized that IGF1R signaling pathway could regulate the outgrowth of sprouting fibers because previous reports strongly suggest that IGF1R signaling regulates neurite outgrowth in the corticospinal motor [43] and dorsal root ganglion neurons [44] and regeneration of the peripheral nervous system motor neurons [45].

However, the expression of IGF1R at the tip of the sprouting fibers was weak, and anti-IGF1R antibody treatment did not change the frequency of the sprouting fibers in NMJ. This indicated that IGF1R signal inhibition was not involved in the axonal elongation of sprouting fibers in the GC muscle. Immunohistochemical analysis of IGF1R to identify the anti-IGF1R antibody targets showed that IGF1R was unusually highly co-localized with postsynaptic components, as detected by fluorescent dye-conjugated α-bungarotoxin. These results indicated that upregulated IGF-I and II in the GC muscle bound to IGF1R at the postsynapse, regulating NMJ regeneration. As expected, all postsynaptic and some presynaptic components were affected by IGF1R inhibition.

IGF1R is expressed in Pax7^+^-SCs, and IGF-I is involved in SC proliferation and differentiation in vitro and in vivo [17, 46]. In addition to its essential role in muscle regeneration, recent evidence suggests that SCs may be the primary regulator of NMJ regeneration [47]. BTX injection also induces SC proliferation in the extraocular muscles 2 weeks after treatment, in which muscle atrophy does not occur even after BTX treatment [48]. In this study, neither induction of SC proliferation nor upregulation of differentiation to MyoD^+^ cells occurred 2 weeks after BTX administration. However, at 6 weeks, anti-IGF1R antibody administration increased the number of Ki67^+^ Pax7^+^ double-positive cells without affecting the number of MyoD^+^ cells. This increase could be attributed to a compensation mechanism independent of IGF1R signaling pathway induced by anti-IGF1R antibody [46]. Therefore, this result also supports the idea that IGF1R inhibition at the postsynapse, specifically downregulates NMJ regeneration. We then asked what the molecular mechanism of this phenomenon is.

Recent evidence suggests that mTOR controls not only translational initiation or autophagy [49] but also neurogenic muscle atrophy [37, 50] or the development and maintenance of NMJ [27, 38]. In Fig. 5D, we organized the main signaling molecules based on the information revealed in the neurogenic muscle atrophy signaling pathway. Moresi et al. [50] reported that neurogenic muscle atrophy was controlled by the signaling pathway of *Hdac4/Myog/*E3 ubiquitin ligases, such as *Fbxo32/MAFbx/Atrogin-1*. Tang et al. [37] revealed that denervation upregulated the mTOR/S6k/translation pathway and activated FoxO by inhibiting Akt in the skeletal muscle. This denervation-induced neurogenic muscle atrophy signaling pathway was well-conserved in our experiments. The results of both studies by Moresi et al. [50] and Tang et al. [37] support the finding that mTOR/S6k/translation, *Hdac4/Myog/Atrogin-1*, and FoxO signaling pathways were activated. However, as shown in Fig. 5A, B, interestingly, autophagy was not activated, in contrast to the result of the study by Castets et al. [38]. This was probably because BTX administration caused minimal or reversible damage in the NMJ [8], different from the degenerated NMJ observed in other injury models [51]. Moreover, when the IGF1R antibody was administrated into the mice with BTX, the antibody only affected translation initiation signaling pathways in our study.

The balance between the synthesis and degradation of synaptic proteins is tightly regulated at the motor endplate [5, 27]. In this study, synaptic genes, such as *Chrna1*, *Chrnd*, *MusK*, or agrin receptor *Lrp4*, are highly upregulated after denervation by BTX. These synaptic genes are regulated by *Hdac4* and *Myog* [50]. Similar to that observed in other nerve injury models [52] or the BTX injection model [53], *Hdac4* and *Myog* were upregulated, but not *Trim63/MuRF1*, the downstream target of FoxO in our BTX injection model. Muscle atrophy signaling pathways have been well-documented [54], and one of the atrogenes (i.e., *Fbxo32/MAFbx32/Atrogin-1*) was significantly activated by BTX treatment in this study. However, this upregulation is not affected by anti-IGF1R antibody treatment in vivo. These results suggested that suppression of translation initiation is specifically affected by IGF1R inhibition.

Currently, BTX treatment has been established for conditions with excessive muscle contraction, such as various forms of dystonia, and esthetic medicine [1, 2]. Furthermore, recent evidence suggests that it could be applicable for preventing migraine [55] or analgetics of arthralgia [56]. However, botulinum therapy has short-term effectiveness and thus requires repeated administration. To avoid induction of neutralizing antibodies against BTX, low-dose injection of BTX is desirable [3, 4]. Therefore, decreasing the number of injections and the total dose of BTX by prolonging its effect by anti-IGF1R antibody would be significantly advantageous in clinical practice.

IGF1R antibodies or inhibitors had been mainly developed for cancer treatment; however, nearly all of them failed owing to their side effects, such as hyperglycemia due to high sequence homology with the insulin receptor [9]. However, teprotumumab, an anti-IGF1R antibody, has been found to improve the outcomes of refractory thyroid ophthalmopathy [25, 57]. Therefore, if clinically approved, teprotumumab could be used for local NMJ control with fewer side effects, because the administration of anti-IGF1R antibody itself did not induce paralysis in the intact muscle.

We also report that IGF1R inhibition could be a beneficial treatment not only for NMJ control, but also for neurodegenerative diseases. Previous studies have shown that neuroinflammation could be attenuated by IGF1R inhibition in an experimental autoimmune encephalomyelitis model of multiple sclerosis [26] or Alzheimer’s disease [58–60].

This study indicated potentially interesting scientific findings, however, two limitations existed. The first limitation was the silencing method of IGF1R. This study achieved partial IGF1R inhibition by antibody administration into GC muscle, however, conditional knockout of IGF1R in postsynapse is ideal. The second limitation was that it is still unclear how the translation signaling pathway contribute to the NMJ regeneration of BTX treated muscles.

In conclusion, anti-IGF1R antibody administration prolongs the effects of BTX by regulating the mTOR/S6 kinase signaling pathway and NMJ regeneration. The IGF1R signaling inhibition could be highly beneficial in clinical practice.

### Reporting summary

Further information on research design is available in the Nature Research Reporting Summary linked to this article.

## Supplementary information

supplemental Figure 1

supplemental Figure 2

Reporting Summary

## Data Availability

Data presented in this study are available from the corresponding authors upon request. Source data were provided as a source data file.

## Code availability

The code used for the morphological analysis of the NMJ has been described previously [34].
