# Supplementary figures and images for: Blocking insulin-like growth factor 1 receptor signaling pathway inhibits neuromuscular junction regeneration after botulinum toxin-A treatment
Source: Cell Death Dis. 2023 Sep 16;14(9):609. doi: 10.1038/s41419-023-06128-w (PMC10505167; doi:10.1038/s41419-023-06128-w)

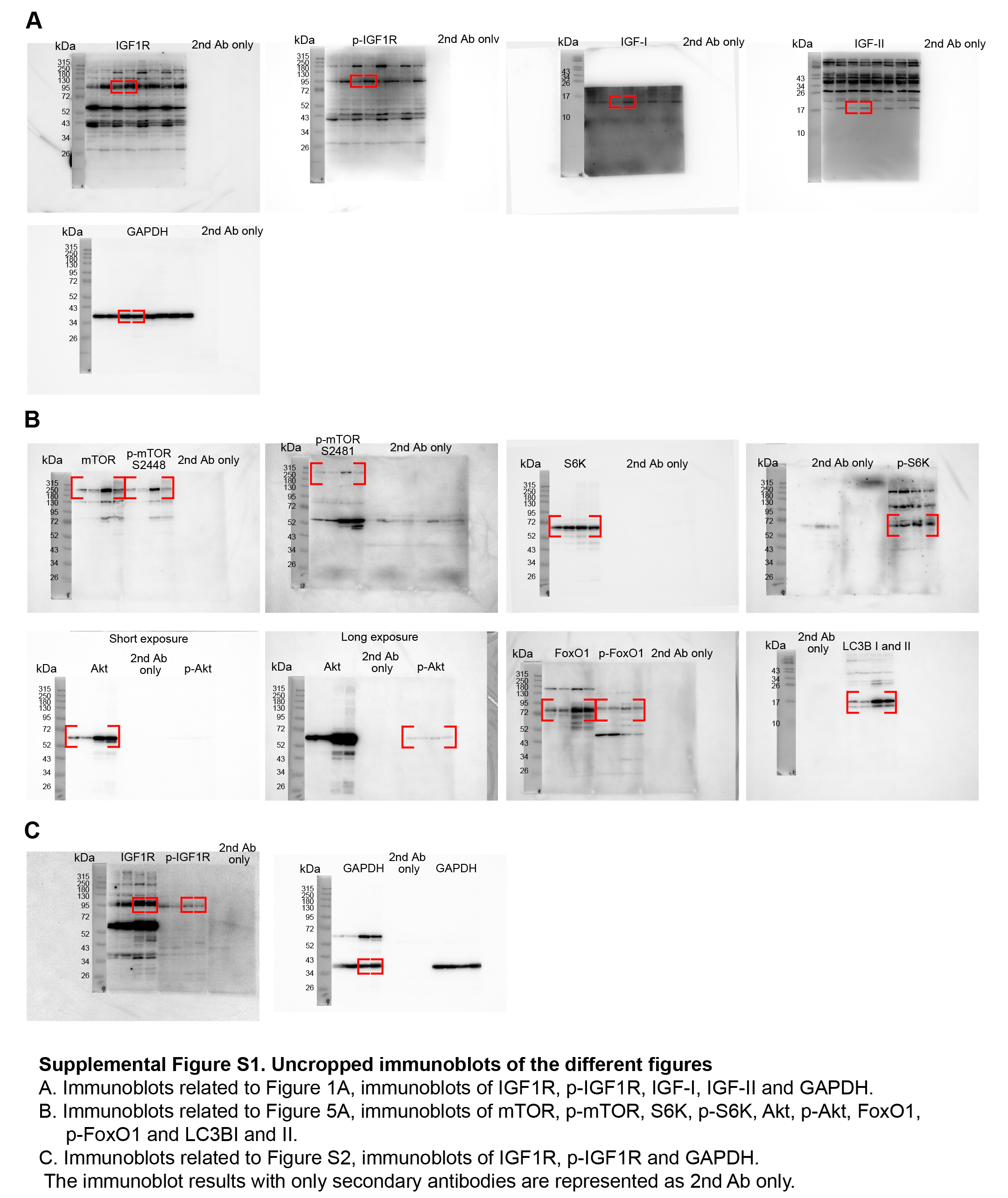

Supplement: Supplementary file 1 — supplemental Figure 1 [file 41419_2023_6128_MOESM1_ESM.tif]

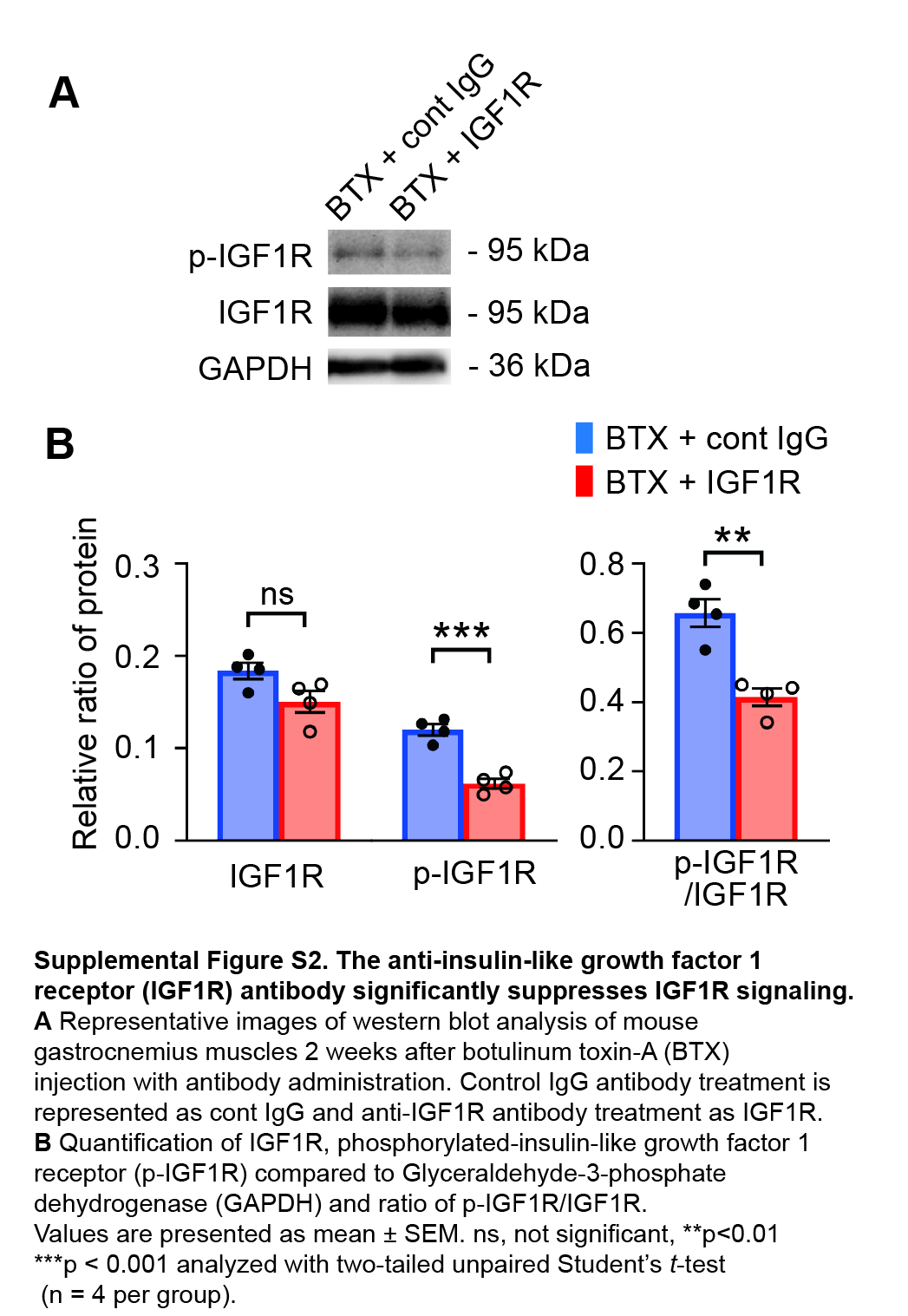

Supplement: Supplementary file 2 — supplemental Figure 2 [file 41419_2023_6128_MOESM2_ESM.tif]
